# Gut fungal profiles reveal phylosymbiosis and codiversification across humans and nonhuman primates

**DOI:** 10.1371/journal.pbio.3003390

**Published:** 2025-09-22

**Authors:** Emily P. Van Syoc, Andres Gomez, Emily R. Davenport, Seth R. Bordenstein

**Affiliations:** 1 One Health Microbiome Center, Huck Institutes of the Life Sciences, The Pennsylvania State University, University Park, Pennsylvania, United States of America; 2 Department of Biology, The Pennsylvania State University, University Park, Pennsylvania, United States of America; 3 Department of Animal Science, University of Minnesota Twin Cities, Minneapolis, Minnesota, United States of America; 4 Department of Entomology, The Pennsylvania State University, University Park, Pennsylvania, United States of America; Johns Hopkins University Bloomberg School of Public Health, UNITED STATES OF AMERICA

## Abstract

Fungi in the gut microbiome, collectively known as the mycobiome, are a prevalent yet neglected component of the human holobiont. A major question in the study of gut microbial communities is whether fungi exhibit eco-evolutionary patterns that are consistent with partner fidelity and long-term associations. We compared gut fungal profiles across natural populations of humans and nonhuman primates and identified significant degrees of primate-mycobiome phylosymbiosis as well as human-enriched fungal taxa. Notably, subsets of fungi are cophylogenetic and exhibit cospeciation patterns in hominids. These findings cautiously originate a new view on the eco-evolutionary potential that can shape the composition of human and primate gut mycobiomes.

## Abstract 2

Fungi in the gut microbiome (the "mycobiome") are a neglected component of the human holobiont. This study of gut fungal profiles across natural populations of humans and non-human primates reveals significant fungal cospeciation patterns in hominids.

## Introduction

The human gut teems with diverse microorganisms that significantly influence health, physiology, and evolution [1–5]. Among these organisms, gut fungi remain underexplored due to assumptions that they are low in biomass relative to bacteria and temporally more variable [6–8]. These suppositions extend to speculation that gut fungi are transiently derived from diet rather than by intimate associations with hosts [8]. However, emerging research indicates that the diversity and structure of gut fungal communities—the mycobiome—link to human aging, health, and disease [9–12]. Beyond the community level, individual fungal species can contribute to gut health and disease, including both protection against infections by activating T helper and antibody-producing cells [13] and contributions to mucosal inflammation [14–16]. Gut fungal implications in modulating gut health and disease warrant a deeper understanding of fundamental drivers of mycobiome assembly.

There is a paucity of eco-evolutionary research on the human mycobiome that hinders development of a broader perspective. Indeed, cornerstone advancements in eco-evolutionary influences on gut bacterial microbiomes [2–4,17–19] have not been rigorously investigated for fungi. These include phenomena such as codivergence, phylosymbiosis, and out-of-Africa migration events that assess the patterns and processes shaping microbial community assembly [2,20,21]. To investigate the human mycobiome in the context of primate evolutionary history, we compiled Internal Transcribed Spacer region 2 (ITS2) data from 183 gut fungal samples in seven free-ranging social groups of nonhuman primates (NHP) and two nonindustrialized human populations. These groups span the Hominidae (humans and great apes) [22], Cercopithidae (cercopithecoids; Old World monkeys) [23], and Indriidae (lemur) [24] families.

We address 3 key questions in the study: (i) Does the primate gut mycobiome exhibit phylosymbiosis? (ii) Which gut fungi are enriched in humans relative to NHP? (iii) Do gut fungi codiversify and/or cospeciate with hominids? Together, results initiate new eco-evolutionary results on the human gut mycobiome and contribute to a framework of gut fungi assembly across our closest evolutionary ancestors.

## Results

### Phylosymbiosis in the primate gut mycobiome

We first sought to position the human gut mycobiome in an evolutionary context by investigating phylosymbiosis, which predicts that (i) host clades harbor distinguishable microbial communities and (ii) host phylogeny recapitulates the microbial community relationships based on ecological beta diversity differences [25,26]. Thus, phylosymbiosis occurs when more closely related hosts species harbor more similar microbiomes. It is distinct from cospeciation and codivergence that specifically refer to symbiotic events involving one type of microorganism [25], which we examine below. Analyses were based on 99% Operational Taxonomic Units (OTUs) from amplified sequences in the same ITS2 region in nonindustrialized humans (*n* = 33), three species of apes (*n*_*Gorilla beringei* _= 21, *n*_*Gorilla gorilla*_ = 26, *n*_*Pan troglodytes schweinfurthi*_ = 13), three species of cercopithedes (guenons, *n*_*Cercocebus agilis*_ = 11, *n*_*Papio cynocephalus*_ = 40, *n*_*Procolobus gordonum*_ = 27), and a distantly related outgroup of indri (*n*_*Indri indri*_ = 12) (S1–S3 Tables and S1 Fig).

**Fig 1:**
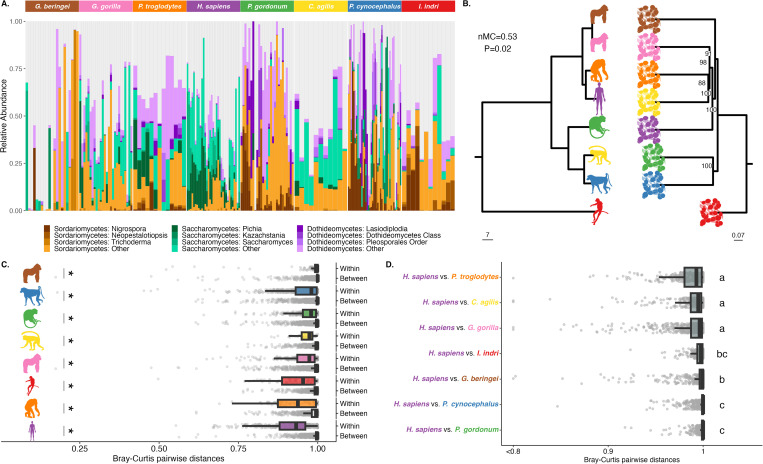
Phylosymbiosis between primates and their gut mycobiomes. **(A)** Sample-wise mycobiome composition for each host species. The most abundant fungal genera and classes (Sordariomycetes, Saccharomycetes, and Dothiomycetes) are shown in colored bars, where the lightest color in the gradient designates the sum of all other genera in that fungal class. Light gray shows all other less abundant fungal genera. **(B)** Primate phylogeny (left; scale bar shows MYA) and fungal Bray–Curtis beta diversity relationships based on 99% OTUs and aggregated by host species (right; shown with bootstrap support for branch placement and scale bar for relative branch lengths). Trees are rooted with the outgroup *Indri indri*. Topological congruency statistics are shown for the normalized Matching Cluster (nMC) metric, where 0 is perfect congruency and 1 is complete incongruency. **(C)** Pairwise Bray–Curtis distances between individual samples for within-host (intraspecific) and between-host (interspecific) comparisons are shown for each host species, ordered by decreasing median of intraspecific distances. Asterisks denote statistical significance (*T* test *P* < 0.05). **(D)** Pairwise Bray–Curtis distances of each human sample to nonhuman primates, ordered by increasing median. Letters denote statistical significance at *P* < 0.05 with Tukey’s post hoc test after ANOVA. Data points that fall below 0.8 (*n* = 7) are shown at ‘<0.8’. The data underlying this figure can be found at https://doi.org/10.5281/zenodo.16749612.

We found at a broad scale that primate hosts harbored distinguishable fungal communities (PERMANOVA *F*_7, 175_ = 2.99, *R*^2^ = 0.11, *P* < 0.001; all pairwise host species PERMANOVA *P* < 0.05, S4 Tables and Fig 1A); and they exhibited significant degrees of phylosymbiosis (Fig 1B). Phylosymbiosis occurs with both Bray–Curtis distances that account for relative abundance patterns (normalized Matching Cluster (nMC) = 0.53, **P* *= 0.02; Fig 1B) and Jaccard’s distance that accounts for OTU detection/absence (nMC = 0.05, *P* = 0.02; S2 Fig). Unlike OTU-based analyses, genus-level comparisons yielded no significant results (S2 Fig), a discrepancy likely stemming from the substantial proportion (47%) of fungal OTUs that could not be classified at the genus level. This common challenge in gut fungi research arises from known reference database limitations [27] and affects the resolution of subtle taxonomic variations, which raises caution when interpreting traditional community diversity analyses. While primates and humans generally clustered within expected host families, the mycobiome profile of *C. agilis* (agile Mangabeys) was more similar to *P. troglodytes* (chimpanzee) than to the other cercopithecoids. This particular placement leads to disruption in the visual congruence for phylosymbiosis.

Phylosymbiosis predicts that microbial community composition exhibits greater similarity within species than between species. While interindividual fungal variability is high, pairwise analyses of beta diversity distances reveal significantly lower average distances within host species compared to between species (Fig 1C, intraspecific versus interspecific Bray–Curtis distances; two-tailed permutation-based test *P* < 0.001, S5 Table). Host species with the most similar mycobiomes were humans (median intraspecific distance = 0.914 ± 0.08), followed by *P. troglodytes* (0.937 ± 0.1), and *I. indri* (0.954 ± 0.1); *G. beringei* were characterized with significantly higher intraspecific distances compared to all other host species (0.998 ± 0.008; ANOVA with Tukey’s post hoc comparisons, all pairwise **P* *< 0.05; S6 Table), as expected and discussed below.

Human mycobiomes were characterized by strong host specificity, demonstrated by the higher intraspecific similarity in Fig 1C and low interspecific similarity (Fig 1D) relative to the NHPs. The lower interindividual variability in human gut mycobiomes compared to the NHPs implied enrichment or depletion of specific fungal taxa relative to evolutionary relatives, as taxon losses in humans are known to frequently occur in gut bacterial communities [28].

### Human-enriched gut fungi

**Fig 2:**
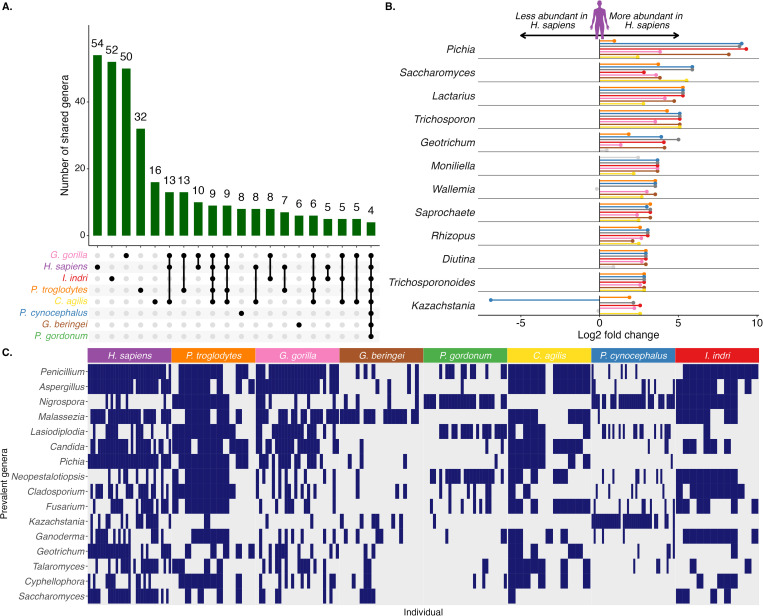
The human gut mycobiome is distinguished from other NHP species by changes in abundant and rare taxa. **(A)** Each host species harbors a unique set of genera, and only 4 are shared across all species. The numbered bar plots correspond to genera present in combinations of host species shown in the dot matrix. Not all combinations (intersections) are shown for brevity. **(B)** Twelve fungal genera differ in relative abundance between humans and at least six of the seven NHP species. Colored points that match the legend in (A) show log2 fold change of each NHP compared to humans, where a positive log2 fold change indicates higher relative abundance in humans. Gray indicates statistical insignificance (FDR-adjusted **P* *> 0.05). **(C)** Prevalent fungal genera in humans, *Pan troglodytes*, *Gorilla gorilla, Cercocebus agilis,* and *Indri indri* are largely absent in the other NHPs. A matrix visualization shows the detection and absence of the most prevalent genera across all host species, where blue shaded squares indicate detection within a sample and unshaded is no detection. The data underlying this figure can be found at https://doi.org/10.5281/zenodo.16749612.

Comparative analyses of fungal taxon differences between nonindustrialized humans and free-range, nonhuman primate gut fungi are valuable for identifying gut commensals that differentiate the human mycobiome. Of the 515 annotated genera in the total dataset, 221 appear in only one host species, and 54 of those are unique to humans (10.5% of all genera; S7 Table; Fig 2A). On average, humans harbor 6 of these (11%) per individual and they comprise 6.5 ± 5.4% of the total relative abundance, while individually, each taxon’s abundance can vary from <0.001% to 15.5% (mean = 1.8%). This proportion of unique genera is similar to *P. troglodytes* (6% of genera are host-specific), *G. gorilla* (10%), and *I. indri* (10%), and higher than the host-specific genera found in *C. agilis* (3%, pairwise Chi-squared test *P* < 0.001), *P. cynocephalus* (2%, *P* < 0.001), *G. beringei* (1%, *P* < 0.001), and *P. gordonum* (0.5%, *P* < 0.001).

To further investigate the specificity of the human gut mycobiome, we determined which prevalent fungal genera differed in relative abundance between humans and NHP host species. Individual models were fit for each fungal genus present in at least 20% of the NHP species and humans (*n* = 205). They were then corrected for multiple comparisons across the entire dataset. Most annotated genera differed between humans and at least one NHP (*n* = 173 of 205; linear model of log2 fold change with False Discovery Rate (FDR)-adjusted **P* *< 0.05; S3 Fig). Of those, 12 fungal genera were strongly associated with human gut mycobiomes and differed between humans and at least six of the seven NHP species (Fig 2B and S7 Table). Almost all these genera were relatively higher in human mycobiomes compared to NHP. These include *Pichia* and *Saccharomyces*, which are both well-established, facultatively anaerobic, health-associated commensals of human gut mycobiota; they comprise a large fraction of human mycobiomes globally [6,9,29,30]. *Lactarius* is an edible mushroom that is a common food item in the human communities of this study [22]. Of the most prevalent genera across all host species, *Saccharomyces*, *Candida*, and *Kazachstania* are more often detected in human samples (Fig 2C). Together, these enrichments of shared and unique human gut taxa indicate that humans differ from other primates both by the presence and abundance of dominant and rare taxa.

### Cophylogeny between gut fungi and the Hominidae family

Cophylogenetic patterns between hominids and individual microbial taxa provide evidence for partner fidelity that can be driven by consistent environmental acquisition, social transmission, or cospeciation. We constructed phylogenies of ITS2 amplicon sequences within each fungal genus and then subset these trees into OTU-level sub-phylogenies across the hominid species. OTUs were clustered at 97% to retain higher sequence diversity, and the sub-phylogenies were statistically compared to the hominid tree using methods for host-bacteria cophylogeny [28,31].

**Fig 3:**
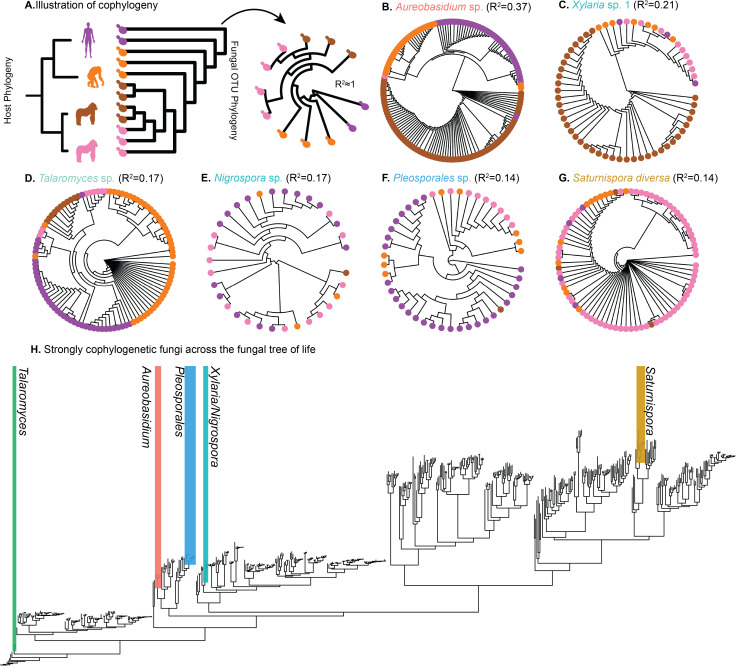
Cophylogenetic signal occurs between diverse gut fungal taxa and hominids. **(A)** Schematic of phylogenies exhibiting codivergence between hominids and an illustrative fungal species, which is then visualized in circular format. In the example, phylogenetic congruence between host and fungi is high (PACo *R*^2^ ≈ 1). **(B–G)** Neighbor-joining trees of fungal OTUs that display cophylogeny with hominid hosts, ordered by decreasing PACo *R*^2^. **(H)** A schematic of the fungal tree of life [39] with highlighted taxa in which cophylogenetic signals (*R*^2^ > 0.1) were detected, colored by each OTU. Species from the genera *Xylaria* and *Nigrospora* in the Xylariales family, are shown in the same region. *N*_*Pan troglodytes*_ = 14; *n*_*Gorilla gorilla *_= 24; *n*_*Gorilla beringei*_ = 20; n_*Homo sapiens*_ = 27. The data underlying this figure can be found at https://doi.org/10.5281/zenodo.16749612.

Eleven of the total 45 OTUs represented in all four hominid host species met significance thresholds and demonstrated a moderate cophylogenetic signal with hominid phylogeny after FDR correction [32,33] (Figs 3A–3G and S4). The OTU with the strongest cophylogeny was *Aureobasidium* sp. (PACo *R*^2^ = 0.37, *Q* < 0.001; Fig 3B), a stress-tolerant, facultative anaerobe previously detected in stool samples of wild *G. gorilla* [34]*,* captive *Macaca mulatta* (rhesus macaques) [35], and humans [36]. OTUs representing the genera *Talaromyces*, *Xylaria*, *Nigrospora*, *Pleosporales*, and *Saturnispora* displayed significant cophylogeny (*R*^2 ^> 0.1, Fig 3C–3G), while two additional *Nigrospora* species, one additional *Xylaria* species (termed ‘*Xylaria* sp. *2*’ for clarity), and *Malassezia arunalokei* had statistically significant but weak clustering with the hominid phylogeny (*R*^2^ < 0.1, S4A–S4E Fig). We observed similar signals when examining cophylogeny in OTUs clustered at 99% similarity (S5 Fig). All OTUs but *Malassezia* belong to the Ascomycota phylum (*Malassezia* is a Basidiomycota; Fig3H) which dominates host-associated mycobiomes [6,9,37]. The noncophylogenetic OTUs with exact sequence variants from all four hominid species are listed in (S8 Table).

**Fig 4:**
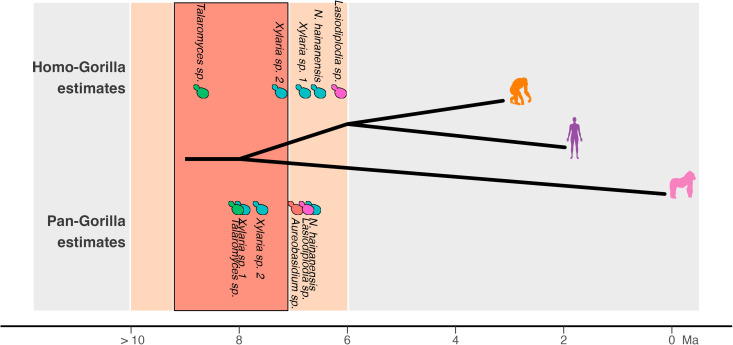
Cophylogenetic fungi with time-calibrated sequence divergences match hominid divergence times. Nucleotide sequence divergence of cophylogenetic fungi was calibrated to the *Homo*-*Pan* split (6 Ma), which was then used to estimate the *Homo*/*Pan*-*G*. *gorilla* split (7.1–9.2 Ma), shown with fungal icons. Estimated speciation times fall within the expected 7.1–9.2 Ma (dark orange) or 6–10 Ma (light orange) Fungal icon colors correspond with Fig 3. Times are shown for the *Homo*-*G. gorilla* split (top) and *Pan*-*G. gorilla* split (bottom). The data underlying this figure can be found at https://doi.org/10.5281/zenodo.16749612.

Next, we evaluated if the cophylogenetic patterns are compatible with cospeciation by testing if fungal nucleotide divergence levels were proportionate to hominid divergence times [20]. We first calculated nucleotide sequence divergence in each cophylogenetic fungi and calibrated it to the *Homo-Pan* split at 6 Ma [38] following previous methods for gut bacteria [20] and since there are no clocks for these specific fungal taxa in host-associated environments. We then tested if the time-calibrated fungal sequence comparisons aligned with known divergence times for the split between *G. gorilla* and the ancestor of *Homo/Pan* (7.1–9.2 Ma [40]). Indeed, the sequence divergence of *Talaromyces* and *Xylaria* sp*.* 2 dated the *Homo-Gorilla* and *Pan-Gorilla* splits within the estimate ranges (Fig 4). Additional cophylogenetic fungi whose sequence divergence matched at least one of the splits with *Gorilla* included *Aureobasidium* sp. (7.0 ± 1.4 Ma **Pan*/*Gorilla**, 5.3 ± 08 **Homo*/*Gorilla**), *Xylaria* sp*.* 1 (7.9 ± 4.0 Ma **Pan*/*Gorilla**, 6.8 ± 3.3 Ma **Homo*/*Gorilla**), and *Nigrospora hananensis* (6.6 ± 2.7 Ma **Pan*/*Gorilla**, 6.5 ± 2.4 Ma **Homo*/*Gorilla**) (Fig 4 and S9 Table). All 11 cophylogenetic fungi expectedly overestimated the *G. gorilla-G. beringei* split (0.15–2.5 Ma) [40] because of the large microbial diversity in *G. beringei* putatively due to ecological drivers (see Discussion). The time-calibrated analysis is cautiously consistent with select events of fungal cospeciation in the Hominidae family.

## Discussion

Gut fungi are ubiquitous yet neglected members of the human gut microbial community. Their eco-evolutionary origins remain a fundamental gap in knowledge after decades of focused study on gut bacteria and hominid evolution [2,20,41]. Our results from free-ranging, NHP and nonindustrialized human communities represent a step toward understanding these forces in the context of well-established themes of ecological and stochastic assembly of fungi [22].

Topological congruency between primates and gut mycobiome relationships statistically support phylosymbiosis using multiple beta diversity metrics and 99% OTUs for fine-scale resolution of fungal taxa. This OTU-level cutoff retains community diversity estimates that are obscured in phylosymbiosis analyses when aggregating fungal data at higher taxonomic levels (e.g., genera), due to limitations in taxonomic annotation of fungi [12,41,42]. Indeed, there was a large proportion of unannotated fungal species and genera (47% in this case) that prohibited treating ITS2 amplicon sequencing data analogously to bacterial 16S data [12,43,44]. Similarly, amplicon sequence variants are not recommended for short-read ITS data [43,44], which is why we use OTU-level analyses throughout. Advancing taxonomy comprehensiveness for fungal marker sequencing will be important for analyses of fungal community structure.

Phylosymbiosis is a significant association between host phylogenetic relationships and host-associated microbiome community relationships. Since it may arise from stochastic and/or deterministic forces, various factors shape the pattern. Primates from natural populations harbored distinguishable gut mycobiomes that clustered intraspecifically and mirrored primate phylogeny to a degree using abundance and presence/absence metrics for beta diversity. However, there were two exceptions in which phylosymbiosis was not perfect. First, the gut mycobiomes of mangabeys (*C. agilis*) and chimpanzees (*P. troglodytes*), which diverged from a common ancestor more than 20 Ma, were placed together in hierarchical clustering. *C. agilis* and *P. troglodytes* samples were collected from different countries, yet the hosts have largely generalist and diverse dietary behaviors, with strong preferences for fruits and seeds [45,46]. Indeed, correspondence between mangabeys and chimpanzees superseding phylogenetic constraints have also been shown with the fecal bacteriome [47], supporting the hypothesis that similar dietary/ecological drivers may trigger microbiome convergence in distantly related NHPs. The second case was that of *G. beringei*, which have highly divergent gut mycobiomes across indviduals. *G. beringei* is the most folivorous species in the dataset and exhibit a distinguishing repertoire of morphological, microbial, and behavioral adaptations specialized in metabolizing plant structural polysaccharides (e.g., leaves and bark) [48,49]. The conflicting drivers of phylogenetic relatedness with diet, geography, gut physiology, and other ecological forces are well documented for gut bacteria [41,47,50,51], and rarely does phylosymbiosis appear as perfect topological congruency in complex mammalian gut systems [25]. Resolving geographic influences on fungal environmental sources and diversity is another future direction of this work. Additionally, lower richness of gut fungal communities compared to gut bacteria could obscure distinguishing phylogenetic signal from noise. Nevertheless, detection of nonrandom gut fungal assembly across primates is the first documentation of phylosymbiosis in gut fungi within mammals.

Phylosymbiosis may arise from a mix of ecological and evolutionary influences including cophylogeny, in which lineages of bacteria diversify in parallel with lineages of their hosts. Among the modestly prevalent taxa at the genus levels, we found that 11 out of 45 OTUs (24%) displayed a signal of cophylogeny with hominids, and this value parallels estimates in bacterial communities [28]. Importantly, a portion of the cophylogenetic fungi are not only topologically congruent with primate evolution; they also demonstrate a striking temporal concordance. Molecular clock analyses of select fungal lineages indicated that fungal speciation events are approximately synchronous with those observed in hominid hosts. This dual evidence—phylogenetic congruence and synchronized speciation timing—suggests the evolutionary trajectories of the microbe and its hominid hosts may be intertwined.

With little precedent in the field, we first analyzed cophylogeny with 97% OTU similarity, risking false negatives to retain sequence divergence in short-read ITS2 sequences that are characterized by divergent evolutionary mechanisms. We then followed up that analysis with 99% OTUs in the supplementary material. Sequence cutoff and length of the fungal ITS region will be important for future investigations of host-associated mycobiomes to determine their impacts on biological outcomes. Testing cophylogeny in a microbial community where one host harbors many symbionts can result in false positives [52]. To minimize this risk, we followed similar work in gut bacterial communities [28,53], that reduced false positives with statistical correction and made inferences based on effect size (PACo *R*^2^) rather than *P* values [54]. Nevertheless, these cophylogenetic genera are commonly detected in the gut mycobiomes of humans and NHP [24,55,56], but whether gut fungi transiently pass through the gut environment via dietary sources or colonize as permanent gut residents is unresolved [8] and a subject of debate [27].

Cophylogenetic taxa *Aureobasidium*, *Talaromyces*, Pleosporales, and *Saturnispora* were successfully cultured from human stool samples in a recent large-scale effort [27]. The genera with the strongest cophylogenetic signal—*Aureobasidium*—is a ‘ubiquitous’ fungal genus widely present in most environments and highly adapted to stress tolerance; they range from generalists to polyextremophiles [57]. As such, some *Aureobasidium* species are facultatively anaerobic [58] and can tolerate or prefer low pH and high temperature [59]. *A. pullulans* and *A. melanogenum* are further opportunistic pathogens with a proven ability to colonize and reproduce in mammalian systems [60]. Given these factors, we postulate that the true diversity of commensal gut fungi that interact with host physiology is likely much greater than previously speculated [12,27]. The wide diversity within fungal genera makes it difficult to draw conclusions about ecological niche from higher taxonomic levels.

Notably, humans had the highest similarity in intraspecific gut mycobiome communities, so we determined which fungal genera were enriched in nonindustrialized humans compared to NHPs. Twelve fungal genera were differentially abundant between humans and the seven NHP host species, supporting the possibility of a unique or distinguishable human fungal biosphere constructed at least in part from abundance increases in prevalent taxa. The top three fungi that differentiated humans span known dietary substrates for the BaAka and Bantu communities in this dataset, such as the edible mushroom *Lactarius* [22], the gut commensal *Saccharomyces,* and *Pichia* that are commonly used in commercial probiotics [61–63] and found in a wide variety of foods [64]. Thus, dietary interactions likely contribute to the complex assembly of the human mycobiome.

In summary, we report several new lines of evidence that support ecoevolutionary influences on the assembly of the gut mycobiome across humans and primate evolutionary history. These results broadly contextualize the roles of fungi in the gut, a task that is often challenged by the low biomass of gut fungi, strict presumptions of diet-driven fungal assembly, and bioinformatic limitations that lag behind bacterial developments [9,12,43]. Uncovering evidence for phylosymbiosis and cospeciation associations of gut fungi in this set of initial analyses initiates a step toward widening the canonical, two-dimensional, emphasis on the evolution of host-bacteria associations in gut microbiomes. These findings set the stage to decipher the broader forces of fungal assembly in human and primate holobionts [65].

## Materials and methods

### Human research participants

All samples were previously collected/sequenced (S1 Table) and no new sample collection or sequencing was conducted as part of this study. The Pennsylvania State University Institutional Review Board exempted the use of this data from institutional review (STUDY00023406).

### Fungal amplicon sequencing data

Study and sample information is provided in S1–S3 Tables. Sequencing data processing was performed with a bespoke script designed to retrieve raw data from the Sequence Read Archive and process in a uniform way. Raw read quality of short-read ITS2 sequences (~300 bp) was assessed with seqkit [66] and FastQC [67]. Ambiguous bases and primer sequences were removed with cutadapt [68] as implemented in the R package dada2 [68] if they were provided in the original study. The following steps were conducted with VSEARCH v2.23.0 [69]. Quality filtering truncated reads was done at Phred score 20 or below, and reads were removed under 10 bp in length. Forward reads (for paired-end sequencing schemes) and single reads (for single-end sequencing schemes) were dereplicated; and quality-filtered data from all three studies were pooled for OTU clustering. Two OTU clustering strategies were employed depending on if the major question centered around taxonomic annotation quality (phylosymbiosis; 99% OTU similarity) or raw sequence diversity (cophylogeny; 97% OTU similarity). OTUs were clustered at the sequence similarity described above, then de novo chimeras were removed with ‘uchime_denovo’, and taxonomy was assigned with SINTAX using the Unite v9 eukaryotic database [70]. Feature table processing and analysis was conducted in R 4.3.1 using primarily the phyloseq [71], microViz [72], and vegan [73] packages. Nonfungal OTUs were removed, then presumable contaminants or sequencing errors were screened by removing OTUs that appeared in only one sample or fewer than twice in the dataset. The filtered feature table were subsampled to a 2,000 read depth by taking the average of 100 rarefaction iterations using the EcolUtils package [74].

### Phylosymbiosis

Host-specific mycobiome profiles were tested with permutational ANOVA (PERMANOVA; ‘adonis’) in the vegan [73] and pairwiseAdonis [75] R packages. A primate phylogenetic tree was obtained from Timetree5 (timetree.org) and pruned to the eight host species present in the dataset, then re-rooted with *I. indri* as the outgroup. Fungal dendrograms were constructed from the filtered, un-rarefied feature table using QIIME2. Fungal profiles were first grouped by host species and averaged to create a representative mycobiome profile for each host species [41] using the QIIME2 plugin feature-table group. The host fungal profiles were subjected to the QIIME2 beta rarefaction plugin, which takes a jackknife approach to iteratively rarefy the feature table to a specified depth and calculate a beta diversity metric, then averages the beta diversity relationships. The plugin outputs a UPGMA-clustered dendrogram, which was rooted with *I. indri* in Figtree v1.4.4 and internal branches rotated as needed with the ape R package [76]. Topological congruency was used to assess phylosymbiosis as the matching of hominid host phylogeny with fungal community diversity, as implemented in ref [41] using the normalized Matching Cluster (nMC) metric in TreeCMP [77]. Statistical significance was determined by comparing the host phylogenetic tree to 100,000 randomly generated trees to calculate the probability of observing a given congruency score with random chance.

Pairwise Bray–Curtis distances were tested for intraspecificity (difference in means between within-species and between-species distances) and interspecificity (distance in means between specific host species). Intraspecific distances were tested with a two-tailed permutation-based test, where the treatment labels were randomly shuffled 999 times to create a null distribution. A two-tailed *P* value was calculated as the number of times the permuted differences in means were equal to or greater than the observed difference in means. Intraspecific distances were tested via ANOVA with Tukey’s post hoc test for multiple comparisons.

### Human-enriched mycobiomes

Pairwise chi-squared tests were used to determine significance of the proportion of unique genera in each hominid species. Differential relative abundance tests were employed to determine which fungal genera had higher or lesser relative abundance in humans compared to hominid primates. First, prevalent genera were extracted and defined as fungal genera present in at least one of each hominid species. Relative abundance of the prevalent genera was tested with log_2_ transformation and linear models of each fungal genera as implemented in the microViz R package [72]. Individual linear models were constructed for each human-primate comparison (i.e., *Homo sapiens* versus *P. troglodytes*, *H. sapiens* versus *G. gorilla*, and *H. sapiens* versus *G. beringei*) and each fungal genus that was prevalent in at least 20% of the individual host species to ensure the results were not driven by extreme zero inflation. Results were concatenated and all *P* values were corrected for multiple comparisons with FDR. The differential relative abundance visualizations were constructed with rphylopic [78] and ggplot2 [79] and the Venn diagram was constructed with the ggvenn R package (https://yanlinlin82.github.io/ggvenn/).

### Cophylogeny

Cophylogenetic tests were implemented at sub-OTU level using a framework to examine each fungal genus for prevalent OTUs. Previous investigations of cophylogeny in gut bacteria compare bacterial phylogeny at the community level [28,53]; the ITS1 and ITS2 fungal markers resolve fungal phylogeny up to the genus or family level, and a mycobiome-wide phylogenetic tree cannot be confidently constructed with only this marker (*Aspergillus*, *Fusarium*, and *Penicillium* genera were also removed from these analyses because ITS sequences in these taxa cannot be distinguished below genus) [43,44]. Therefore, we constructed a framework to ‘scan’ through the mycobiome, construct a phylogenetic tree for each genus, and investigate OTU-level subtrees for signals of cophylogeny. This was operationalized by obtaining exact sequence variants for each fungal genus that was detected in all four hominid species, dereplicating in VSEARCH [69], and aligning the sequences in MAFFT [80]. Alignments were trimmed with clipkit [81] and neighbor-joining trees were constructed using Jukes-Cantor distance calculations with the ape R package [76]. OTU-level subtrees were extracted from the genus-level trees and filtered to retain subtrees with sequences sampled from all four hominid species. Then, statistical tests for cophylogeny with the hominid phylogenetic tree (described above) were implemented with the parafit [33] method as implemented in the ape R package [76] and the Procrustean Approach to Cophylogeny test as implemented in the PACo R package [32]. Both tests were implemented with the ‘cailliez’ correction for negative eigenvalues [53] and 99 permutations, and PACo additionally used a conservative ‘quasiswap’ null test, which assumes that hosts and microbes do not track each other in evolutionary time [53]. Significant cophylogenetic signals were considered as fungal trees that met FDR-corrected *Q* < 0.05 in both parafit and PACo. PACo *R*^2^ was calculated as (1 − Procrustes sum of squares) [54]. To determine the false positive rate of cophylogeny tests, we randomly shuffled the tip labels of the fungal neighbor-joining trees 100 times and conducted the parafit and PACo tests. We observed a false positive rate of 5% which decreased to 0% with corrections for multiple comparisons implemented the same way as on our dataset, which validates the utility of our dual-analysis approach to reduce false positive results. The visualization of cophylogenetic fungi was made using the ggtree [82] package and the fungal tree of life was obtained from ref [38].

### Nucleotide sequence divergence

Nucleotide sequence divergence (NSD) was compared in the cophylogenetic fungal OTUs using the multiple sequence alignments described above. The multiple sequence alignment was subset to exact sequence variants from each OTU, and the ape R package was used to calculate the NSD proportion between each sequence with the dist.dna function. The average NSD between sequences from *H. sapiens* and *P. troglodytes* was calibrated to the estimated Homo-Pan speciation time of 6 Ma. This calibration was used to scale the NSD between all other hominid species to an estimated speciation time. The visualization of fungal sequence divergence was made with the ggtree [82] and rphylopic [78] packages with R code contributions from ref [83].

## Supporting information

S1 Fig Approximate geographic distribution of the nonhuman primate species. The data underlying this figure can be found at https://doi.org/10.5281/zenodo.16749612.
(DOCX)

S2 Fig Topological congruency tests at the fungal OTU and genus levels with Bray–Curtis and Jaccard distances. Primate phylogeny (left; scale bars show MYA) and fungal beta diversity dendrograms (right; shown with bootstrap support for branch placement and scale bar for relative branch lengths). Trees are rooted with the outgroup *Indri indri*. Topological congruency statistics are shown for the normalized Matching Cluster (nMC) metric, where 0 is perfect congruency and 1 is complete incongruency. Fungal dendrograms are shown at **(A)** the OTU level with Jaccard’s binary distance, and at the aggregated genus level for **(B)** Bray–Curtis, and **(C)** Jaccard’s binary distances, respectively. The data underlying this figure can be found at https://doi.org/10.5281/zenodo.16749612.
(PNG)

S3 Fig Fungal genera that differ in relative abundance between humans and NHP. Colored lines show the log2 fold change between humans and nonhuman primates (NHP), where a positive value indicates higher relative abundance in humans. NHP are colored and ordered by the legend on the right; a gray color indicates statistical insignificance (FDR-adjusted *P *> 0.05) and missing taxon colors indicate a lack of detection or low prevalence of the fungal genus in that NHP. The data underlying this figure can be found at https://doi.org/10.5281/zenodo.16749612.
(PNG)

S4 Fig Cophylogenetic signal occurs between diverse gut fungal taxa and hominids (continued from Fig 3). **(A–E)** Neighbor-joining trees of fungal OTUs that display cophylogeny with hominid hosts, ordered by decreasing PACo R2. **(F)** An expanded schematic of the fungal tree of life depicted in Fig 3H shows all fungal taxa with significant cophylogeny in the main text and supplement. Clade colors match each fungal OTU. *Xylaria* and *Nigrospora*, both members of the Xylariales family, are shown in the same region. The data underlying this figure can be found at https://doi.org/10.5281/zenodo.16749612.
(PNG)

S5 Fig Cophylogenetic signal occurs between hominids and fungal OTUs clustered at 99% similarity. **(A–H)** Neighbor-joining trees of fungal OTUs that display cophylogeny with hominid hosts, ordered by decreasing PACo *R*^2^. The data underlying this figure can be found at https://doi.org/10.5281/zenodo.16749612.
(PNG)

S1 Table Metadata corresponding to each study where ITS data was accessed. (XLSX)

S2 Table Metadata corresponding to each host species where ITS data was accessed. (XLSX)

S3 Table Lookup table for geographic information corresponding to the samples from PRJNA686661. (XLSX)

S4 Table Pairwise PERMANOVA comparisons between host species, showing the effect size (*R*^2^ and *F* statistic) and *P* values for each pairwise comparison. (XLSX)

S5 Table Summary statistics of pairwise intraspecific distances and *P* values from a two-tailed permutation test. (XLSX)

S6 Table Summary statistics of pairwise interspecific distances, tested with ANOVA and Tukey’s post hoc comparisons. (XLSX)

S7 Table Differential relative abundance between each NHP species and humans for prevalent fungal genera, showing summary statistics for log2 linear modeling. (XLSX)

S8 Table Cophylogeny test statistics across all tested OTUs showing parafit and PacCo test statistics and FDR-adjusted *P* values. (XLSX)

S9 Table Estimated hominid speciation based on calibrated fungal sequence divergence. For each hominid ‘split’, estimated divergence times from fungal sequence divergence is shown with 95% confidence intervals.
(XLSX)

## Abbreviations

FDR: False Discovery Rate
ITS2: Internal Transcribed Spacer region 2
NHP: nonhuman primates
NSD: nucleotide sequence divergence
nMC: normalized Matching Cluster
