# Supplementary figures and images for: Gut fungal profiles reveal phylosymbiosis and codiversification across humans and nonhuman primates
Source: PLoS Biol. 2025 Sep 22;23(9):e3003390. doi: 10.1371/journal.pbio.3003390 (PMC12453195; doi:10.1371/journal.pbio.3003390)

**
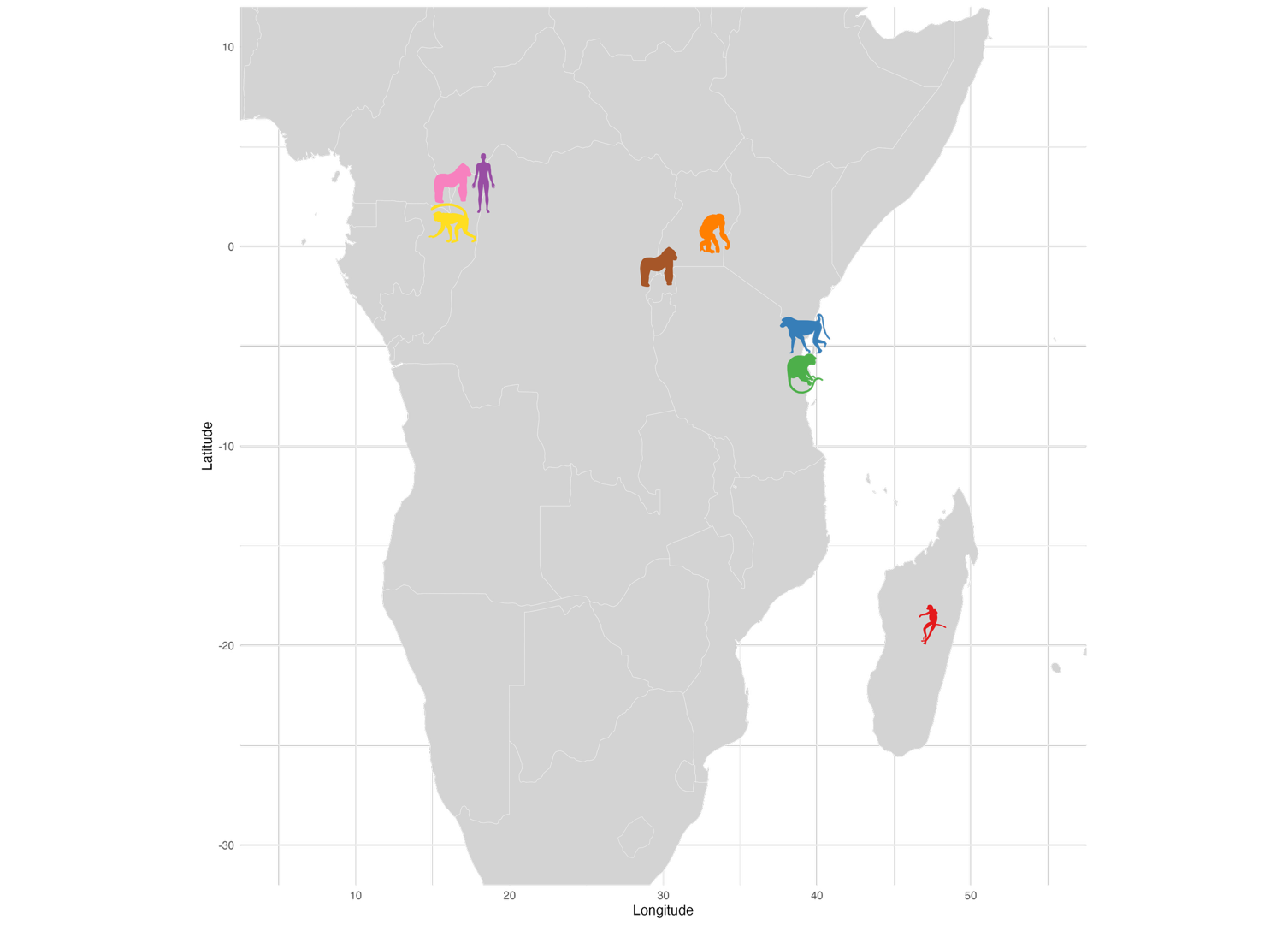

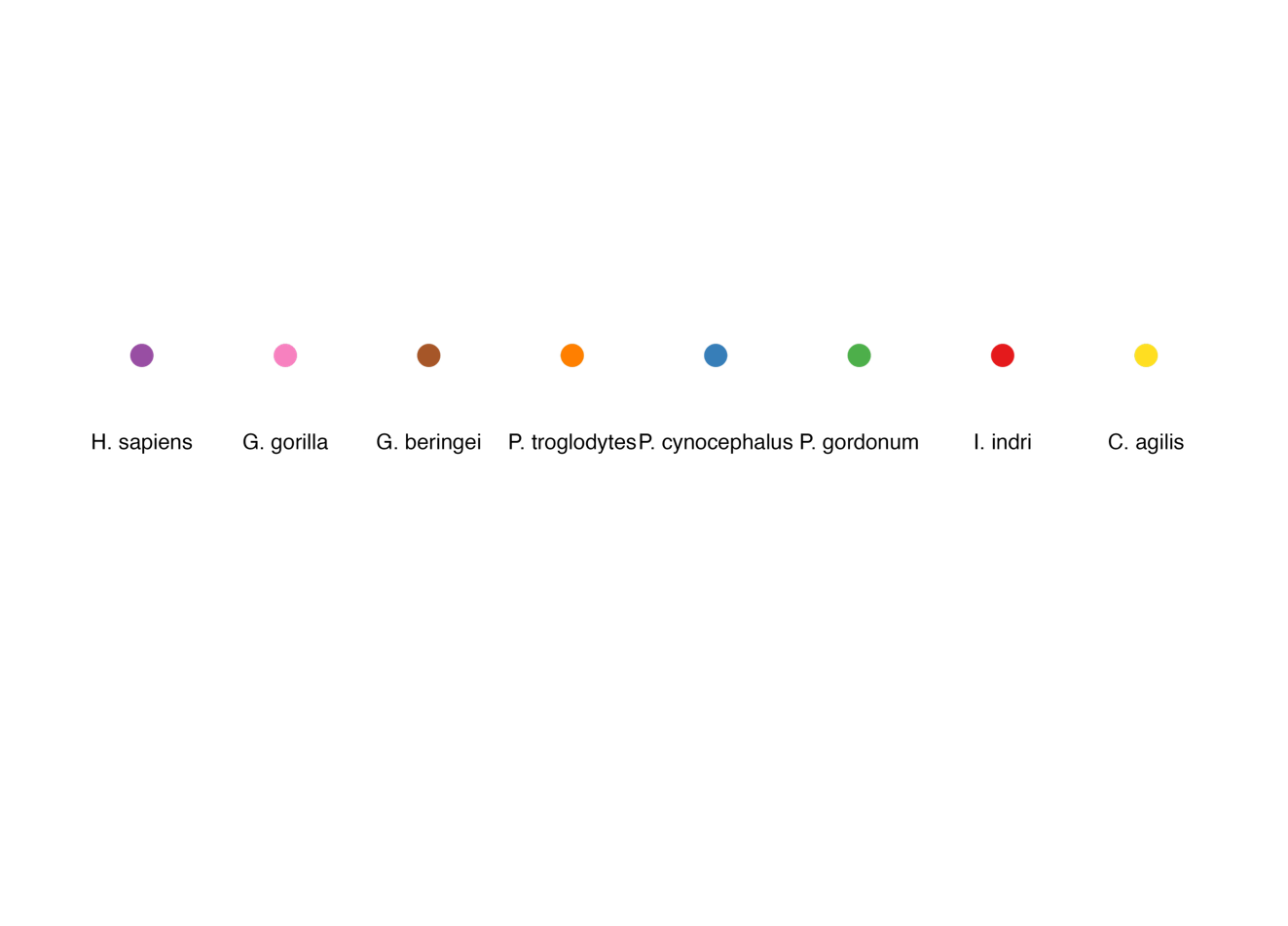
**

Supplement: S1 Fig — The data underlying this figure can be found at https://doi.org/10.5281/zenodo.16749612. (DOCX) [file pbio.3003390.s001.docx]

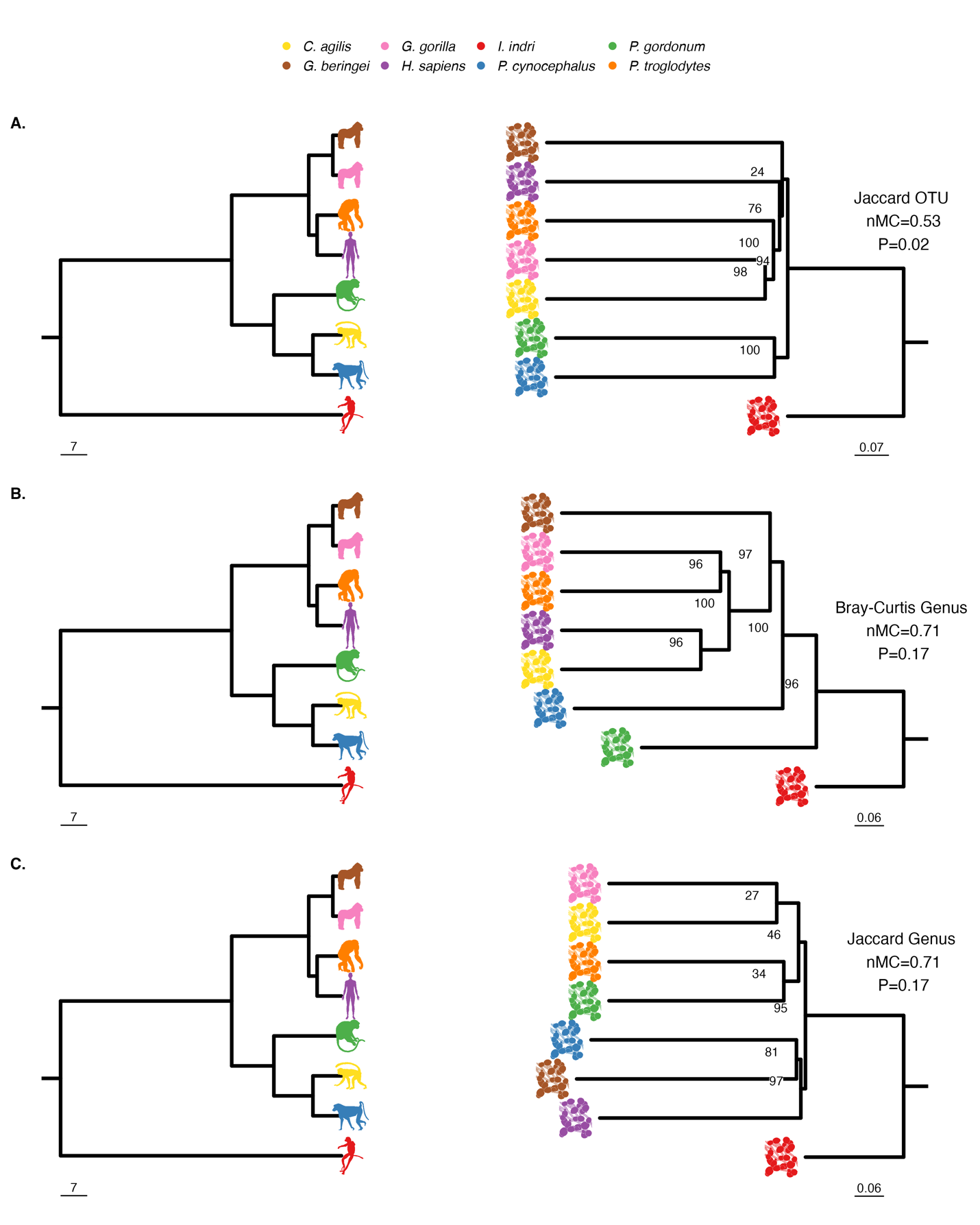

Supplement: S2 Fig — Primate phylogeny (left; scale bars show MYA) and fungal beta diversity dendrograms (right; shown with bootstrap support for branch placement and scale bar for relative branch lengths). Trees are rooted with the outgroup Indri indri. Topological congruency statistics are shown for the normalized Matching Cluster (nMC) metric, where 0 is perfect congruency and 1 is complete incongruency. Fungal dendrograms are shown at (A) the OTU level with Jaccard’s binary distance, and at the aggregated genus level for (B) Bray–Curtis, and (C) Jaccard’s binary distances, respectively. The data underlying this figure can be found at https://doi.org/10.5281/zenodo.16749612. (PNG) [file pbio.3003390.s002.png]

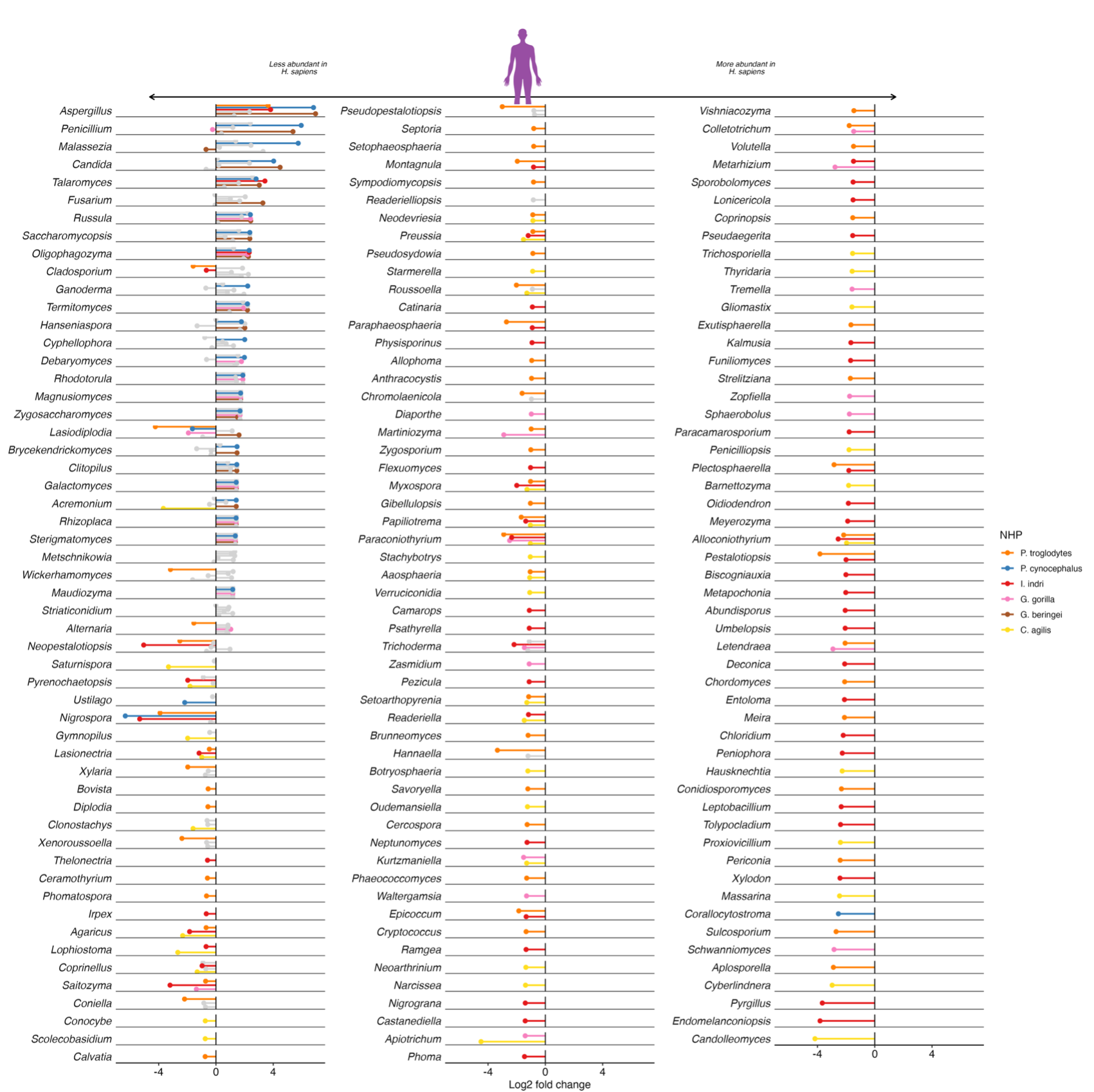

Supplement: S3 Fig — Colored lines show the log2 fold change between humans and nonhuman primates (NHP), where a positive value indicates higher relative abundance in humans. NHP are colored and ordered by the legend on the right; a gray color indicates statistical insignificance (FDR-adjusted P > 0.05) and missing taxon colors indicate a lack of detection or low prevalence of the fungal genus in that NHP. The data underlying this figure can be found at https://doi.org/10.5281/zenodo.16749612. (PNG) [file pbio.3003390.s003.png]

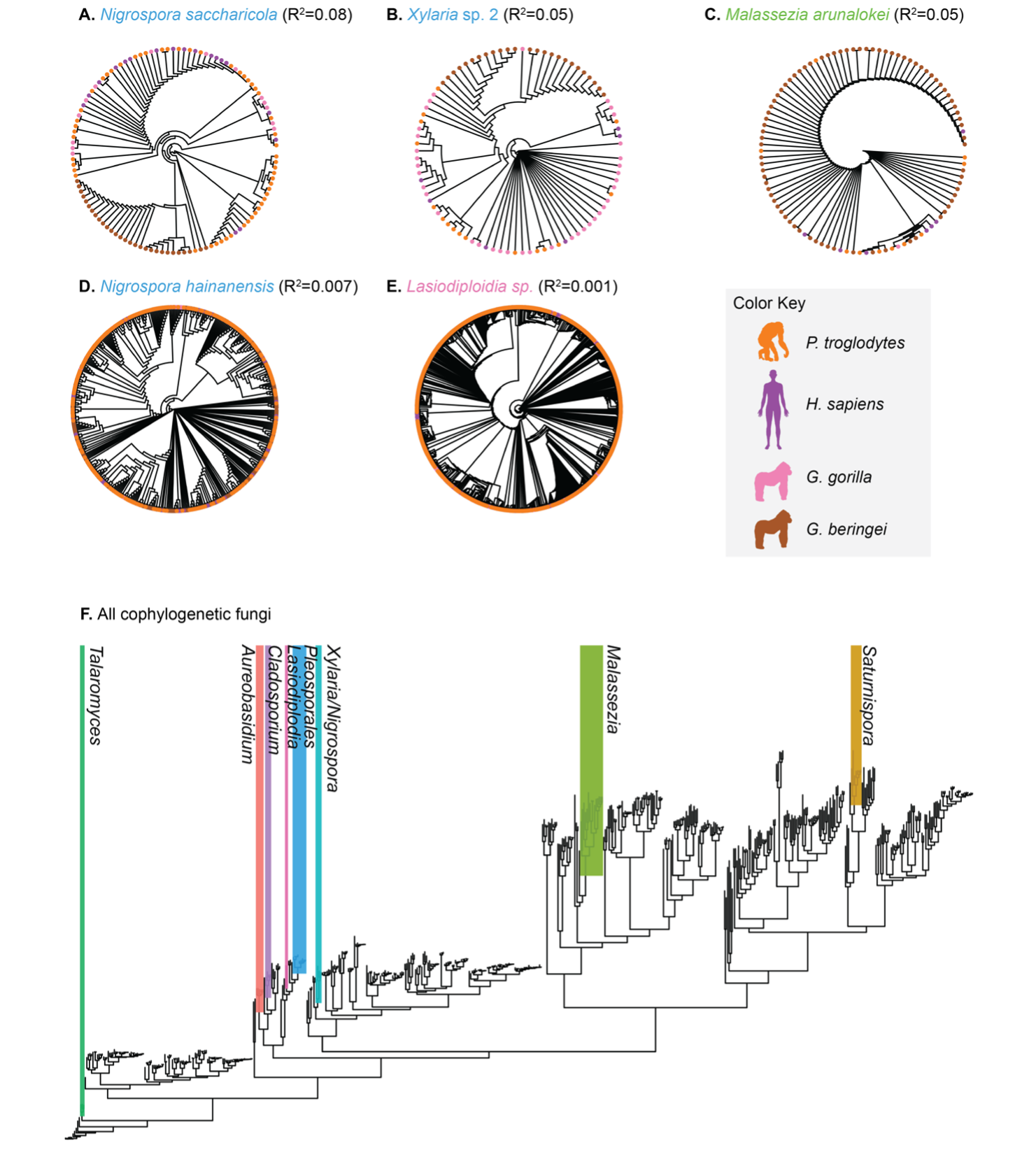

Supplement: S4 Fig — (A–E) Neighbor-joining trees of fungal OTUs that display cophylogeny with hominid hosts, ordered by decreasing PACo R2. (F) An expanded schematic of the fungal tree of life depicted in Fig 3H shows all fungal taxa with significant cophylogeny in the main text and supplement. Clade colors match each fungal OTU. Xylaria and Nigrospora, both members of the Xylariales family, are shown in the same region. The data underlying this figure can be found at https://doi.org/10.5281/zenodo.16749612. (PNG) [file pbio.3003390.s004.png]

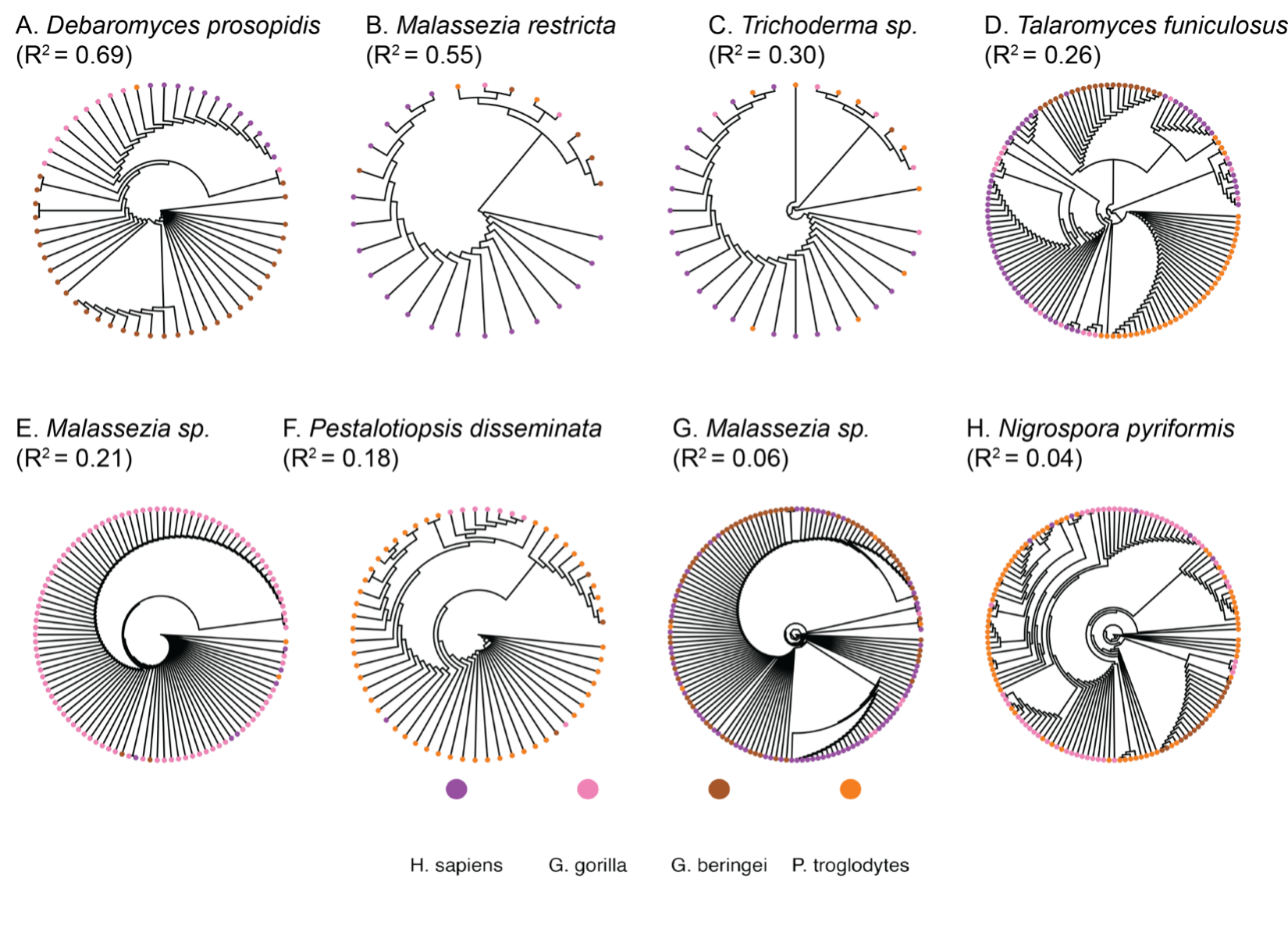

Supplement: S5 Fig — (A–H) Neighbor-joining trees of fungal OTUs that display cophylogeny with hominid hosts, ordered by decreasing PACo R2. The data underlying this figure can be found at https://doi.org/10.5281/zenodo.16749612. (PNG) [file pbio.3003390.s005.png]
